# Assessing the change in diet from baseline to first re-examination – results from the German National Cohort (NAKO)

**DOI:** 10.1186/s40795-026-01454-6

**Published:** 2026-08-19

**Authors:** Nina Wawro, Sven Knüppel, Katharina S. Weber, Wolfgang Lieb, Paula Stürmer, Sabrina Schlesinger, Michael Leitzmann, Beate Fischer, Stefanie Castell, Börge Schmidt, Antje Hebestreit, Gowsiga Loganathan, Tobias Pischon, Katharina Nimptsch, Lilian Krist, Stefan N Willich, Annette Peters, André Karch, Henning Teismann, Nadia Obi, Sylvia Gastell, Stefanie Jaskulski, Matthias B. Schulze, Jakob Linseisen

**Affiliations:** 1 Epidemiology, University of Augsburg, Augsburg, Germany; 2 Institute of Epidemiology, Helmholtz Center Munich, Neuherberg, Germany; 3 Department Food and Feed Safety in the Food Chain, Unit Human Study Centre Consumer Health Protection, German Federal Institute for Risk Assessment, Berlin, Germany; 4 Institute of Epidemiology, University of Kiel, Kiel, Germany; 5 German Diabetes Center, Institute for Biometrics and Epidemiology, Leibniz Center for Diabetes Research at Heinrich Heine University, Düsseldorf, Germany; 6 German Center for Diabetes Research (DZD), Neuherberg, Germany; 7 Department of Epidemiology and Preventive Medicine, University of Regensburg, Regensburg, Germany; 8 Department of Epidemiology, Helmholtz Centre for Infection Research (HZI), Brunswick, Germany; 9 Institute for Medical Informatics, Biometry and Epidemiology, University Hospital Essen, University of Duisburg-Essen, Essen, Germany; 10 Leibniz Institute for Prevention Research and Epidemiology – BIPS, Bremen, Germany; 11 Molecular Epidemiology Research Group, Max Delbrück Center for Molecular Medicine in the Helmholtz Association (MDC), Berlin, Germany; 12 Biobank, Max Delbrück Center for Molecular Medicine in the Helmholtz Association (MDC), Berlin, Germany; 13 Charite - Universitatsmedizin Berlin, corporate member of Freie Universitat Berlin and Humboldt-Universitat zu Berlin, Berlin, Germany; 14 Institute of Social Medicine, Epidemiology and Health Economics, Charité - Universitätsmedizin Berlin, Berlin, Germany; 15 Institute for Medical Information Processing, Biometry and Epidemiology, Ludwig-Maximilians-Universität München, Munich, Germany; 16 Institute of Epidemiology and Social Medicine, University of Münster, Münster, Germany; 17 Institute for Occupational and Maritime Medicine (ZfAM), University Medical Center Hamburg-Eppendorf, Hamburg, Germany; 18 German Institute of Human Nutrition Potsdam-Rehbruecke, Nuthetal, Germany; 19 Institute of Epidemiology and Prevention, Faculty of Medicine and Medical Center, University of Freiburg, Freiburg, Germany; 20 Institute of Nutritional Science, University of Potsdam, Nuthetal, Germany

**Keywords:** Usual intake, NAKO, Macro nutrient intake, Cohort study, Food group intake

## Abstract

**Background:**

In the NAKO cohort, dietary assessment instruments were applied at baseline (2014–2019) and during the first re-examination (2019–2024). The aim was to assess and describe changes in dietary intake over time, and to evaluate the possible consequences of pooling dietary data from both assessment periods for NAKO participants with insufficient or missing dietary information at baseline.

**Methods:**

Full dietary information in both examination waves was collected in a group of 9,035 participants recruited in the study centers in Berlin-South and Freiburg. The mean difference between the baseline and first re-examination visit was 4.8 years (SD = 0.5). For both time periods, the usual intake of food items for each participant was estimated per examination wave using the modified Multiple Source Method (MSM) that incorporated repeated 24-hour food lists (24 h-FL) and a food frequency questionnaire (FFQ), combined with external data from the German National Nutrition Survey II (NVS II). Total energy intake and macronutrient intake were derived by linking food items to the German food composition database. On the individual level, differences in intake estimates were examined and the concordance was assessed.

**Results:**

Overall, the results showed a high concordance of energy and macronutrient intake estimates between both time periods (ICC ≥ 0.70 for macronutrients) and only small mean differences. On average, energy intake decreased by 56 kcal/day in males and 37 kcal/day in females. At the food group level, only small changes were observed, except for the consumption of meat and meat products, as well as milk and dairy products. In females, average meat and meat product consumption decreased from baseline to the first re-examination by 7.3 g/day and in males by 14.6 g/day; the intake of milk and dairy products decreased by about 20 g/day in both females and males.

**Conclusion:**

In this NAKO sub-sample, habitual intake estimates for energy, macronutrients and most food groups were highly consistent between recruitment and first re-examination. While changes in two specific food groups were observed, overall macronutrient and energy intake remained stable over the five-year period. These findings suggest that in case of incomplete dietary data at baseline, it may be appropriate to use data from both examination waves to estimate a person’s usual dietary intake.

**Supplementary Information:**

The online version contains supplementary material available at https://doi.org/10.1186/s40795-026-01454-6.

## Introduction

Various personal and social factors influence the stability of dietary intake in adults. Furthermore, observed differences in dietary intake vary depending on the chosen level of detail during the dietary assessment, such as assessing at the nutrient level or the food group level. Evidence from large cohort studies, including the Framingham study or the Heidelberg part of the European Prospective Investigation into Cancer and Nutrition (EPIC-Heidelberg) study, suggested only moderate agreement in dietary intake over periods of 5 years or more [1, 2]. Methodologically, these studies often used food frequency questionnaires (FFQs), which have known limitations in precision and validity. Thus, observed differences between time periods may reflect both true changes in dietary behavior and measurement errors. A UK Biobank reproducibility study reported only moderate reproducibility for repeated dietary measures over four years, with intra-class correlation coefficients (ICCs) varying by nutrient and food group intake, concluding that apparent long-term instability is partly attributable to dietary assessment imprecision [2]. External data sources, such as survey or sales data, may therefore provide valuable complementary context for interpreting changes in reported dietary intake.

The German National Cohort (NAKO, “NAKO Gesundheitsstudie”) is an ongoing multi-centric, population-based cohort study conducted across 18 study centers in Germany; it was designed to answer research questions about risk factors for the development of non-communicable (NCDs) and also communicable diseases, to develop effective prevention strategies, and to identify new methods for early disease detection [3]. Within the examination program, a novel dietary assessment approach was established [4, 5]. This approach combined two instruments: up to four repeated 24-hour food lists (24 h-FL) and a food frequency questionnaire (FFQ) [6, 7]. It fulfilled the requirement of minimizing the burden on NAKO participants by using the 24 h-FL as a repeated short-term dietary assessment instrument that can be completed in about 10 min. The very strict time limit for the examination program in this cohort study made it necessary to develop a fast to complete 24-hour assessment method [6].

When estimating the consumption probabilities of food items, including additional 24 h-FLs improved the accuracy of the estimated consumption probability; this was shown in the NAKO study [5] and in other studies [8, 9]. With a limited number of 24 h-FLs available, every additional 24 h-FL reduces within-variance, thereby improving the precision of the consumption probability estimate. As the number of completed 24 h-FLs and FFQs were relatively low during the baseline examination, participants were asked to fill in additional 24 h-FL during the first re-examination, notwithstanding the initial study plan. All available 24 h-FL and FFQ from the baseline examination and the first re-examination were included to model consumption probabilities for NAKO participants and thus to derive the usual dietary intake for as many individuals as possible. Consequently, the need to evaluate potential changes in dietary intake on the individual level emerged.

The present study aimed to compare usual dietary intake estimates calculated for both examination waves on the individual level, which was possible for a subgroup of participants in two NAKO study centers (Berlin-South and Freiburg). A secondary aim was to assess the possible consequences of pooling dietary data from both assessment periods for NAKO participants with insufficient or missing dietary information at baseline.

## Methods

### NAKO study

NAKO participants were residents in one out of 16 study regions across Germany and were recruited through a network of 18 study centers. They were identified based on age and sex-stratified samples randomly drawn from compulsory registries of residents within the defined study areas. During the recruitment phase (2014–2019) of the NAKO, a total of 204,706 persons (103,284 females and 101,422 males) aged 19 to 74 years visited the NAKO study centers. A detailed description of the extensive examination program, including an interview, various questionnaires – including those questionnaires for dietary assessment -, medical examinations, and the sampling of bio-specimens can be found elsewhere [3, 10].

This study uses dietary data from participants in the study centers Berlin-South and Freiburg; in both study centers, an almost complete dietary assessment was achieved during both examination waves. Re-examination started in 2019, after the baseline recruitment was finished. A total of 9,035 participants (4,277 participants from Berlin-South and 4,758 participants from Freiburg) were included in the analysis. For both time periods at least one 24 h-FL and a FFQ had to be available for a participant to be included in the analysis. The mean interval between the baseline and the first re-examination visit was 4.8 years (SD = 0.5 years, min = 3.1 years and max = 8.7 years).

The German National Cohort (NAKO) was approved by the initial vote of the ethics committee of the Bavarian Medical Association (“Bayerische Landesärztekammer” (BLÄK), protocol code 13023), followed by all local ethics committees of the study sites. The study was conducted in accordance with the Declaration of Helsinki of 1975 (in the current, revised version). Written informed consent for the participation in data and biospecimen collection and physical examinations was obtained from all participants. The NAKO study was registered in the German Clinical Trials Register (DRKS) under the identifier DRKS00037328 on 11.9.2025 (https://www.drks.de).

### Dietary assessment

Dietary intake was assessed at both baseline and the first re-examination using 24 h-FLs and an FFQ. The 24 h-FL collected dietary information on the dietary intake during the past 24 h across 27 food groups without quantifying portion sizes. Participants completed the first 24 h-FL at the study center and up to three subsequent 24 h-FL at home, one every three months after an invitation by the study center (exact days were randomly determined). Long-term intake over the past 12 months was assessed using the FFQ [7]. Participants reported consumption frequency for a closed list of items with assigned portion sizes, with options ranging from “never” to “daily intake”. Supplement use and recent dietary changes were also assessed. Both electronic tools included automated completion checks that prompted participants to fill in unanswered questions prior to submission.

### Usual dietary intake estimation

Following the approach of the modified Multiple Source Method (MSM) as described by Wawro, Gastell [5], for the current analysis, the consumption probability was modelled separately for each examination wave, only including questionnaires with a sufficient completeness of > 70%, completed during the respective examination wave. For every participant, two estimates for the consumption probability of every single food item were derived, one for each examination wave.

We estimated the usual dietary intake for each food item as the product of consumption probability and the person-specific amount consumed on consumption days. When modelling the consumption probability based on the repeated measurements from up to four 24 h-FLs, covariates for adjustment (age, biological sex, body mass index (BMI), education level and smoking status) and the FFQ information for the relevant item were included in the models. The FFQ information was especially important for the estimation of rarely consumed food items or food items that were not consumed at all. As in the whole cohort, the person-specific consumption amounts were estimated using a prediction model based on data from the German National Nutrition Survey (NVS II) by transferring parameter estimates to the NAKO data set: linear mixed effects models were applied to estimate individual consumption-day amounts based on two repeated 24-hour dietary recalls of the NVS II, adjusted for age, sex, BMI, education, and smoking status [5]. Usual intakes of the single food items were summed to form main food groups and subgroups, according to the EPIC-Soft food groups scheme [11] that was developed within the EPIC (European Prospective Investigation into Cancer and Nutrition) study. The full list of food items and their grouping according to the EPIC-Soft classification can be found in the Supplementary Table S1 (Food items categorised according to the EPIC-Soft main food groups) in [5]. The single food items were linked to the German food composition database [12]. Nutrients were summed up for every individual across all food items.

Covariates included in the probability models were assessed at baseline and limited to a small number of most important variables to ensure consistency when using the same variables in the prediction models of the consumption amounts from the NVS II [13]. We assumed that any changes in the covariates BMI, smoking status or education level by the time of the follow-up occurred at random and did not affect the estimation of the usual dietary intakes. Inclusion of time-specific covariates would have led to computational challenges, although it is beyond all questions that all participants aged from baseline to re-examination. Age (years) was computed as the date of baseline examination minus the date of birth. Sex (male/female) was reported in the main baseline questionnaire. Education was grouped into three categories, representing low, moderate and high education levels. Grouping was based on the highest self-reported education level (ISCED 2011) by combining information on school and vocational training as no degree, in training or vocational education (level 0 to 4, i.e. low), professional school or similar degree (level 5 & 6, i.e. moderate), university degree or higher (level 7 & 8, i.e. high). The body mass index (BMI, kg/m^2^) was calculated from measured body weight and height. Height was measured using the Seca Stadiometer 274, and weight was measured with the Seca Body Composition Analyzer (mBCA) 515. All participants were measured in their underwear. BMI was calculated as weight (kg) divided by height in meters squared (m^2^). BMI was grouped into underweight (BMI < 18.5), normal weight (18.5 ≤ BMI < 25), overweight (25 ≤ BMI < 30) and obese (BMI ≥ 30). Smoking status was categorized as never, former, or current smoker.

The basal metabolic rate (BMR) was calculated according to the age and sex-specific formulas provided by Schofield [14]. Using the ratio of total energy intake (TEI) to BMR, under-reporters were identified as TEI/BMR < 0.8, which represented the 5% quantile. The group of high energy intake reporters was defined as TEI/BMR > 1.6, which represented the 95% quantile.

### Statistical methods

To assess the change in usual dietary intake between the baseline and the first re-examination, we compared mean intake values from both time periods for total energy intake, macronutrients, and the main food groups. The distribution of individual differences in dietary intake between baseline and the first re-examination (intake in g/day at re-examination minus intake in g/day at baseline) is characterized by reporting standard deviation (SD), minimum, maximum, and 10-, 25-, 50-, 75- and 90%-percentiles.

To assess the concordance of individual estimates, we provide the Spearman rank correlation, Pearson correlation and intraclass correlation coefficient (ICC, using R library irr 0.84.1, type=’agreement’, where a value > 0.7 indicated good agreement [15]). We provide both correlation coefficients as scatterplots suggested no strict linear relationships for all food groups.

To illustrate the agreement of estimates between the different time periods, scatterplots stratified by the pattern of available 24 h-FLs for the main food groups are provided. The pattern is named according to the number of available 24 h-FL in both assessment waves, e.g. Pattern 2_4 represents two available 24 h-FLs at baseline and four available 24 h-FLs at first re-examination. Bland-Altman plots for the intake of total energy and macronutrients are provided for men and women separately, to examine individual differences with respect to mean intake amounts.

As sensitivity analyses, we examined the changes among plausible reporters (excluding miss-reporters) in diet, as defined by a TEI to BMR ratio between 0.8 and 1.6. In addition, analyses were restricted to subjects with four 24 h-FLs and an FFQ available in both examination waves.

Analyses were carried out using R 4.3.1. Usual intake estimation was carried out using SAS 9.4. We followed STROBE-nut reporting guidelines [16].

## Results

The study population comprised 9,035 participants, 48% males and 52% females, of which 53% were examined at the study center in Freiburg and 47% at the study center in Berlin-South (Table 1). At baseline, the mean age was 51.1 ± 11.9 years for both sexes; the mean BMI in males was 26.2 ± 4.0 kg/m^2^ and in females 24.8 ± 4.8 kg/m^2^.

**Table 1:** Population characteristics for the total sample and stratified by sex at baseline. Mean, standard deviation (sd) for continuous variables, number (n), % for categorical variables

		Total (*n* = 9,035)		Males (*n* = 4,333)		Females (*n* = 4,702)	
		mean	sd	mean	sd	mean	sd
Age		51.1	11.9	51.3	12.0	51.0	11.9
BMI		25.4	4.5	26.2	4.0	24.8	4.8
		**n**	**%**	**n**	**%**	**n**	**%**
BMI groups	underweight	118	1.3	14	0.3	104	2.2
	normal weight	4,574	50.6	1,806	41.7	2,768	58.9
	overweight	3,154	34.9	1,903	43.9	1,251	26.6
	obese	1,189	13.2	610	14.1	579	12.3
Centre	Freiburg	4,758	52.7	2,326	53.7	2,432	51.7
	Berlin-South	4,277	47.3	2,007	46.3	2,270	48.3
Education level	low	3,121	34.5	1,172	27.0	1,949	41.5
	medium	2,713	30.0	1,454	33.6	1,259	26.8
	high	3,201	35.4	1,707	39.4	1,494	31.8
Smoking status	never	4,483	49.6	2,074	47.9	2,409	51.2
	current smoker	1,294	14.3	620	14.3	674	14.3
	former smoker	3,258	36.1	1,639	37.8	1,619	34.4
Pattern of 24 h-FL	1 _ 1	63	0.7	41	0.9	22	0.5
	1 _ 2	100	1.1	48	1.1	52	1.1
	1 _ 3	28	0.3	18	0.4	10	0.2
	1 _ 4	85	0.9	45	1.0	40	0.9
	2 _ 1	194	2.1	111	2.6	83	1.8
	2 _ 2	252	2.8	112	2.6	140	3.0
	2 _ 3	84	0.9	37	0.9	47	1.0
	2 _ 4	245	2.7	107	2.5	138	2.9
	3 _ 1	81	0.9	52	1.2	29	0.6
	3 _ 2	82	0.9	43	1.0	39	0.8
	3 _ 3	48	0.5	25	0.6	23	0.5
	3 _ 4	166	1.8	74	1.7	92	2.0
	4 _ 1	745	8.2	367	8.5	378	8.0
	4 _ 2	813	9.0	421	9.7	392	8.3
	4 _ 3	544	6.0	280	6.5	264	5.6
	4 _ 4	5,505	60.9	2,552	58.9	2,953	62.8

Pattern of 24 h-FL A_B: number of 24 h FL at baseline (A)_number of 24 h-FL at re-examination (B)

More than 60% of the participants filled in four 24 h-FLs during the baseline examination wave as well as during the first re-examination wave. In addition, about 23% had completed four 24 h-FLs during the baseline but less during the first re-examination. All other patterns of available 24 h-FLs were observed below 3% (Table 1).

Tables 2 and 3 describe the mean intake values for total energy, energy-providing nutrients, and the main food groups during the baseline and during the first re-examination stratified by sex. Intake estimates were derived adjusted for age, sex, BMI, education level and smoking status. Additionally, the distribution of individual differences of estimates between both time periods and the concordance of measurements are presented. In both, males and females, a slight decrease in mean energy intake was observed (56 kcal/day (SD = 264 kcal/day) and 36 kcal/day (SD = 164 kcal/day), respectively), which was reflected in slightly decreased intake of protein, fat, carbohydrates, and alcohol. Mean dietary fiber intake increased by 0.8 g/day in both, males and females, from baseline to first re-examination. The minimum and maximum values of the difference between males and females revealed extreme changes in diet. For example, an increase in total energy intake estimates of more than 1,000 kcal/day between the two measurement time periods may be understood as extreme. At the macronutrient level, we observed a good accordance of individual intake estimates of at least 0.70 in both sexes for the ICC. Correlation coefficients were even slightly higher for all macronutrients than the ICCs.

**Table 2:** Total energy intake, macronutrients and main food groups in males (*N* = 4,333). Mean and standard deviation (sd) of intake estimates at baseline examination, re-examination and individual differences (re-examination – baseline). Characterization of difference distribution by min, max and 10, 25, 50, 75 and 90%-percentiles. Concordance of intake estimates (Spearman correlation coefficient, Pearson correlation coefficient and Intraclass correlation coefficient (ICC))

	Baseline		Re-Examination					Difference Re-Examination - Baseline								
	mean	sd	mean	sd	Spearman	Pearson	ICC	mean	sd	min	max	q10	q25	q50	q75	q90
Total energy intake [kcal/d]	2,168	379	2,113	399	0.77	0.77	0.76	-55.9	264.1	-1,460.3	1,161.3	-377.0	-216.4	-49.8	107.5	263.9
Alcohol [g/d]	11.8	9.1	11.4	9.5	0.81	0.79	0.79	-0.4	6.0	-64.3	36.4	-6.6	-2.9	-0.3	2.1	5.7
Fiber [g/d]	24.7	6.8	25.5	7.7	0.78	0.78	0.77	0.8	4.9	-20.3	35.6	-4.8	-2.0	0.5	3.4	6.5
Protein [g/d]	76.0	14.1	73.1	14.7	0.71	0.72	0.70	-2.9	10.8	-69.4	51.9	-15.9	-9.5	-3.0	3.6	10.2
Fat [g/d]	91.3	17.5	89.6	18.5	0.73	0.73	0.73	-1.8	13.2	-67.9	61.4	-17.7	-9.6	-1.5	6.2	13.7
Carbohydrates [g/d]	234.5	47.6	228.2	49.2	0.76	0.77	0.76	-6.3	33.0	-165.3	164.4	-47.6	-26.5	-6.2	14.1	33.3
Potatoes [g/d]	54.3	15.3	53.6	14.8	0.48	0.48	0.48	-0.7	15.3	-92.5	79.9	-18.1	-9.0	-0.6	7.6	16.7
Vegetables [g/d]	167.9	54.3	168.8	60.0	0.70	0.70	0.70	0.8	44.6	-263.8	326.2	-50.5	-26.1	-0.1	25.8	54.3
Legumes [g/d]	7.4	4.1	7.7	4.6	0.51	0.49	0.48	0.3	4.4	-31.0	32.6	-4.2	-1.7	0.0	2.3	5.0
Fruits and nuts [g/d]	263.3	114.2	276.0	125.0	0.74	0.74	0.74	12.5	86.1	-527.3	489.1	-86.9	-37.5	7.5	57.6	116.9
Dairy products [g/d]	223.6	112.3	203.0	111.0	0.63	0.64	0.62	-20.4	95.2	-612.0	424.0	-137.8	-72.1	-14.9	34.1	87.2
Cereals [g/d]	197.2	45.0	195.6	46.4	0.67	0.68	0.68	-1.6	36.3	-204.8	197.5	-46.6	-23.4	-1.5	21.1	41.6
Meat and meat products [g/d]	96.8	42.1	82.2	42.8	0.73	0.75	0.70	-14.6	30.2	-233.5	197.3	-49.6	-30.6	-12.9	1.4	18.3
Fish and shellfish [g/d]	20.6	13.5	19.8	14.8	0.62	0.57	0.57	-0.7	13.2	-105.7	146.4	-13.1	-6.4	-0.8	3.7	11.9
Egg and egg products [g/d]	19.1	11.2	20.7	12.9	0.52	0.54	0.52	1.6	11.7	-65.2	92.6	-9.4	-4.0	0.8	6.2	13.7
Fats [g/d]	20.8	6.5	20.6	6.8	0.69	0.71	0.71	-0.2	5.1	-20.8	26.4	-6.5	-3.0	0.0	2.9	5.7
Sugar and sweets [g/d]	41.3	18.7	39.4	18.8	0.66	0.68	0.68	-1.9	15.0	-90.3	62.5	-20.1	-10.7	-1.7	7.1	16.2
Cake [g/d]	47.9	19.5	46.4	20.0	0.62	0.64	0.64	-1.6	16.7	-121.1	134.4	-19.7	-10.5	-1.8	7.1	16.4
Non-alcoholic drinks [g/d]	2,015.9	373.4	1,968.7	386.7	0.68	0.69	0.69	-47.2	298.0	-1,649.8	1,482.8	-406.0	-218.4	-44.9	127.8	315.2
Alcoholic drinks[g/d]	206.3	161.5	196.5	165.5	0.79	0.76	0.76	-9.8	112.6	-943.5	713.2	-127.8	-55.3	-5.6	37.5	108.1

**Table 3:** Total energy intake, macronutrients and main food groups in females (*N* = 4,702). Mean and standard deviation (sd) of intake estimates at baseline examination, re-examination and individual differences of re-examination - baseline. Characterization of difference distribution by min, max and 10, 25, 50, 75 and 90%-percentiles. Concordance of intake estimates (Spearman correlation coefficient, Pearson correlation coefficient and Intraclass correlation coefficient (ICC)

	Baseline		Re-Examination					Difference Re-Examination - Baseline								
	mean	sd	mean	sd	Spearman	Pearson	ICC	mean	sd	min	max	q10	q25	q50	q75	q90
Total energy intake [kcal/d]	1,571	289	1,533	300	0.78	0.78	0.78	-37.1	193.5	-889.5	839.0	-274.7	-155.6	-35.7	82.0	193.9
Alcohol [g/d]	5.4	4.7	5.2	5.0	0.78	0.73	0.73	-0.3	3.5	-42.0	33.9	-3.6	-1.7	-0.2	0.9	3.0
Fiber [g/d]	21.7	5.9	22.5	6.6	0.76	0.76	0.75	0.8	4.4	-34.9	25.3	-4.3	-1.8	0.6	3.1	6.1
Protein [g/d]	54.0	10.2	52.4	10.7	0.73	0.73	0.72	-1.5	7.6	-39.2	33.7	-10.5	-6.1	-1.6	3.0	7.5
Fat [g/d]	66.1	13.9	65.4	14.3	0.75	0.74	0.73	-0.6	10.3	-52.6	49.0	-12.8	-6.7	-0.6	5.1	11.5
Carbohydrates [g/d]	175.4	36.2	169.7	37.5	0.76	0.76	0.75	-5.7	25.5	-171.5	137.4	-36.2	-20.8	-5.7	9.5	24.5
Potatoes [g/d]	41.6	11.9	43.0	12.3	0.49	0.50	0.49	1.4	12.1	-80.2	60.6	-12.4	-5.0	1.2	8.4	15.6
Vegetables [g/d]	184.6	56.0	188.6	60.3	0.68	0.70	0.69	4.0	45.3	-257.2	297.6	-50.8	-24.8	3.5	31.3	59.0
Legumes [g/d]	7.3	4.1	8.0	5.0	0.50	0.49	0.47	0.7	4.7	-24.6	32.3	-4.1	-1.4	0.2	2.7	5.8
Fruits and nuts [g/d]	296.9	114.6	302.7	123.3	0.70	0.68	0.68	5.8	94.8	-619.9	557.2	-100.4	-48.8	2.0	56.8	117.4
Dairy products [g/d]	199.5	84.6	180.2	85.3	0.58	0.60	0.58	-19.3	76.5	-467.3	375.4	-109.7	-62.1	-15.7	27.0	70.5
Cereals [g/d]	125.1	32.0	123.6	33.1	0.70	0.70	0.70	-1.5	25.2	-157.4	135.4	-31.6	-16.7	-1.2	14.4	28.2
Meat and meat products [g/d]	43.9	22.4	36.5	22.0	0.77	0.76	0.71	-7.3	15.4	-143.5	81.2	-24.9	-14.9	-6.3	0.2	8.3
Fish and shellfish [g/d]	15.0	9.5	14.9	11.0	0.60	0.52	0.52	-0.1	10.1	-80.0	64.8	-10.1	-4.8	-0.5	3.3	9.8
Egg and egg products [g/d]	14.8	9.0	15.9	9.8	0.52	0.52	0.52	1.1	9.2	-62.1	72.7	-7.6	-3.2	0.7	4.9	10.3
Fats [g/d]	12.5	3.8	12.6	3.9	0.67	0.69	0.69	0.1	3.0	-13.7	12.4	-3.7	-1.8	0.1	1.9	3.8
Sugar and sweets [g/d]	30.1	13.2	27.6	13.3	0.63	0.64	0.62	-2.5	11.3	-73.2	45.5	-16.3	-9.0	-2.3	3.9	10.6
Cake [g/d]	37.3	14.1	36.0	14.1	0.62	0.61	0.61	-1.3	12.4	-128.4	117.3	-14.9	-7.9	-1.2	5.2	12.0
Non-alcoholic drinks [g/d]	2,076.2	360.6	2,046.9	369.5	0.69	0.70	0.70	-29.3	283.3	-1,389.4	1,554.3	-373.8	-199.2	-31.3	139.7	320.0
Alcoholic drinks[g/d]	69.2	59.2	65.2	62.9	0.77	0.72	0.72	-4.0	45.6	-511.1	467.0	-47.2	-22.0	-3.4	12.3	39.6

For the main food groups, analogous results were obtained, with the median values of the differences being close to zero. For males, a higher mean usual intake was observed for fruit and nuts (12.5 g/day, 5% increase compared to baseline) during re-examination. In contrast, a lower mean usual intake was estimated for dairy products (-20.4 g/day, 9% decrease), meat and meat products (-14.6 g/day, 15% decrease), and non-alcoholic drinks (-47.2 g/day, 5% decrease) at re-examination. The mean change in usual intake for all other food groups was lower than 10 g/day and lower than 5%. The lowest correlations were observed for potatoes (0.48), legumes (0.51), and egg and egg products (0.52) in males, although the median and mean of the differences were close to 0 g/day. The highest correlation was seen for the usual intake of alcoholic drinks (0.79). For all other food groups, the correlation coefficients were above 0.60 in men (Table 2).

In females, similar results were observed, but they were less pronounced. A higher mean usual intake was observed in fruit and nuts (5.8 g/day, 2% increase) and vegetables (4 g/day, 2% increase) at re-examination compared to baseline. In contrast, a lower mean usual intake was estimated for dairy products (-19.3 g/day, 10% decrease), meat and meat products (-7.3 g/day, 17% decrease), and non-alcoholic drinks (-29.3 g/day,1% decrease) at re-examination. The mean change in usual intake for all other food groups was lower than 4 g/day. As in males, the lowest correlations were observed for potatoes (0.49), legumes (0.50), and egg and egg products (0.52) in females, although the median and the mean of the differences were close to 0 g/day. The highest correlations were seen for the usual intake of alcoholic drinks (0.77) and meat and meat products (0.77). For all other food groups, correlations were above 0.58 in females (Table 3).

**Fig. 1:**
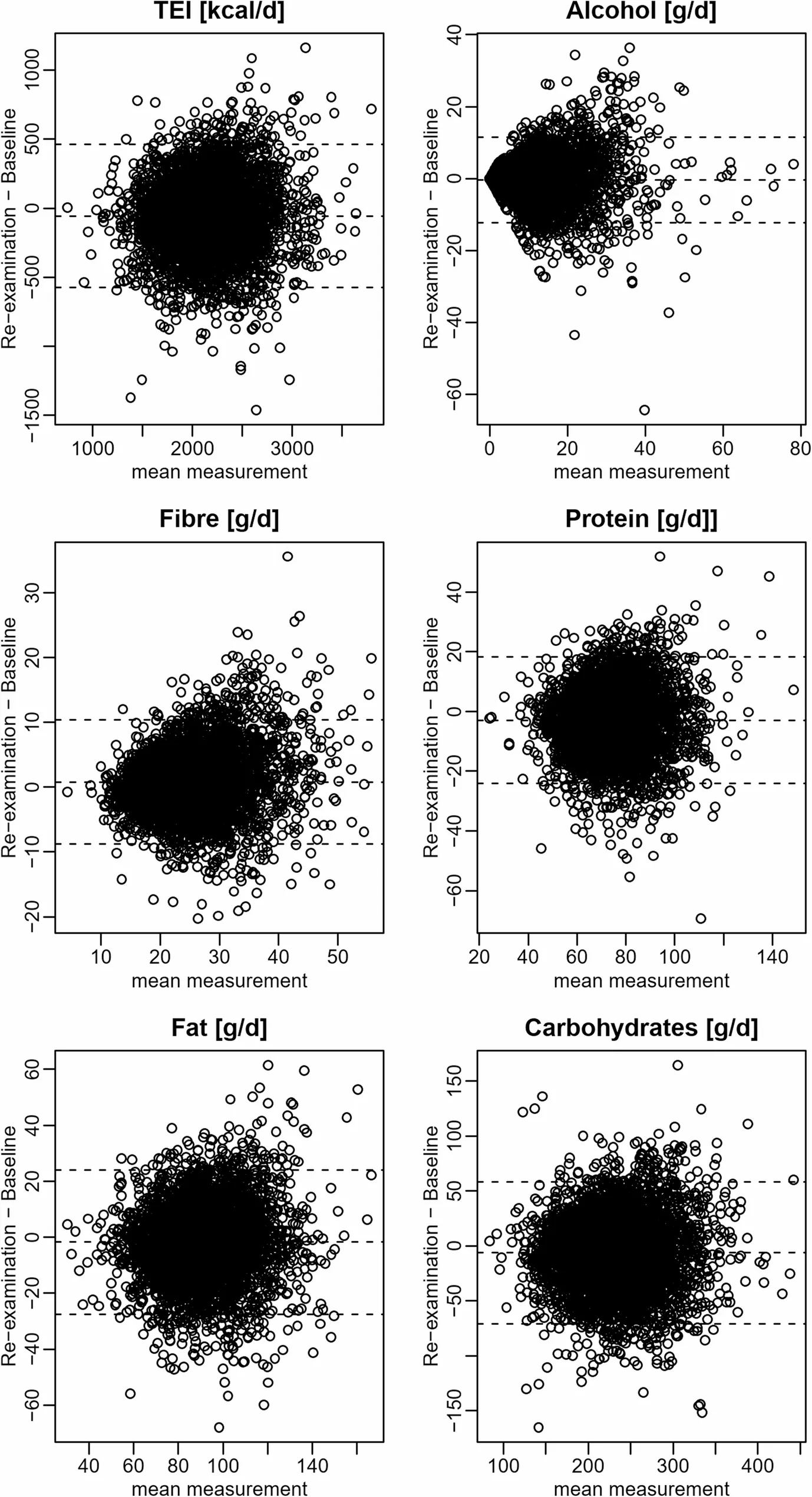
Bland-Altman plots for total energy intake and macronutrients for estimates at baseline and at re-examination in males. Dashed lines represent mean differences ± 1.96 times standard deviations

**Fig. 2:**
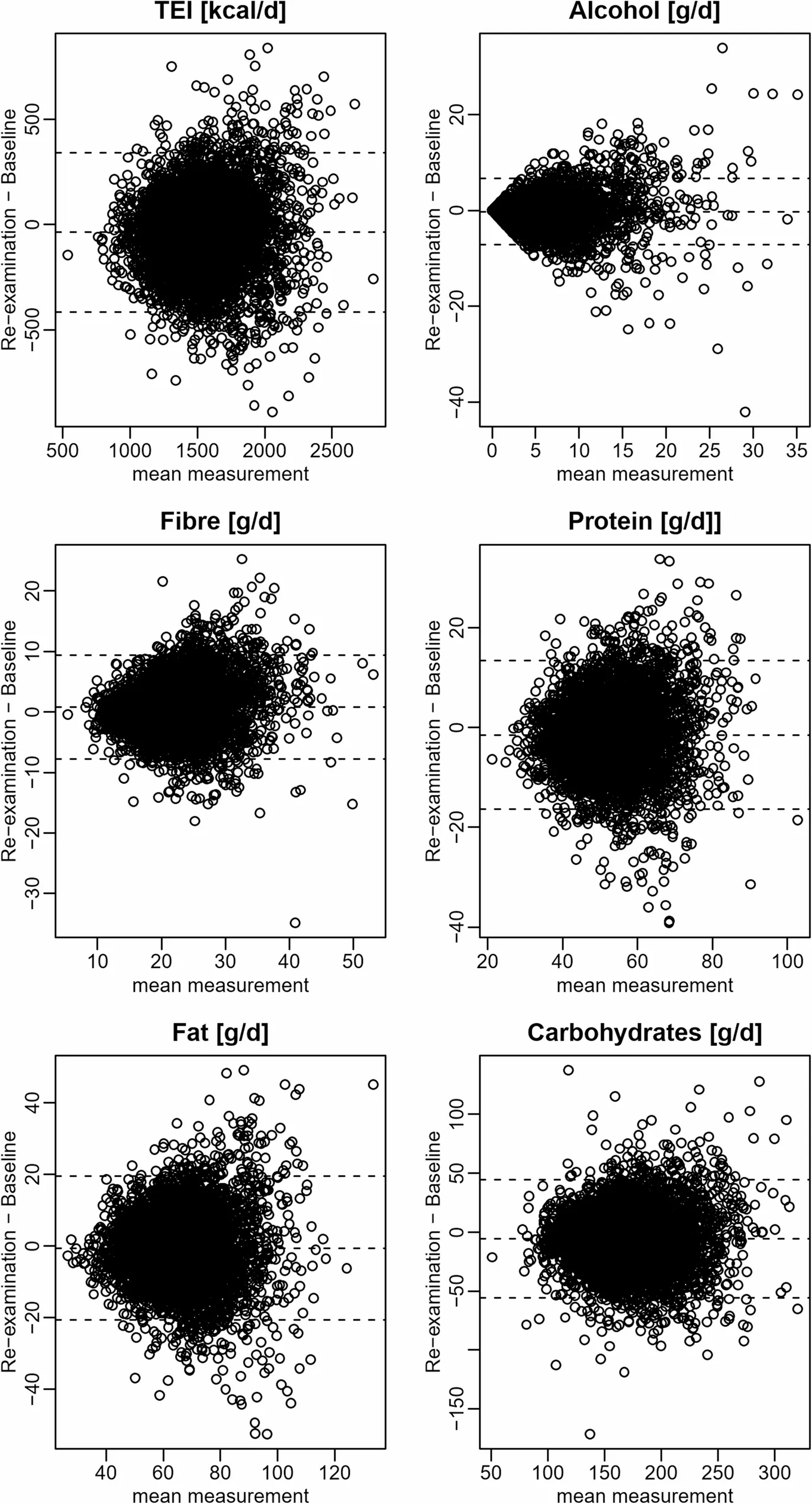
Bland-Altman plots for total energy intake and macro nutrients for estimates at baseline and at re-examination in females. Dashed lines represent mean differences ± 1.96 times standard deviations

The Bland Altman plots for the intake of total energy and macronutrients for males (Fig. 1) and females (Fig. 2) showed little systematic differences for total energy intake, protein, carbohydrates, fiber, and fat intakes. In all plots, outliers, i.e., participants with major changes in the usual diet, can be visually identified. The usual intake of alcohol shows a different picture due to the zero amounts of consumption (non-consumers). Non-consumers in at least one time period imply the straight edge on the left-hand side of the plot. A larger dispersion of the differences (y-axis) was observed for larger mean intake values (x-axis), which were more pronounced in females than in males.

**Fig. 3:**
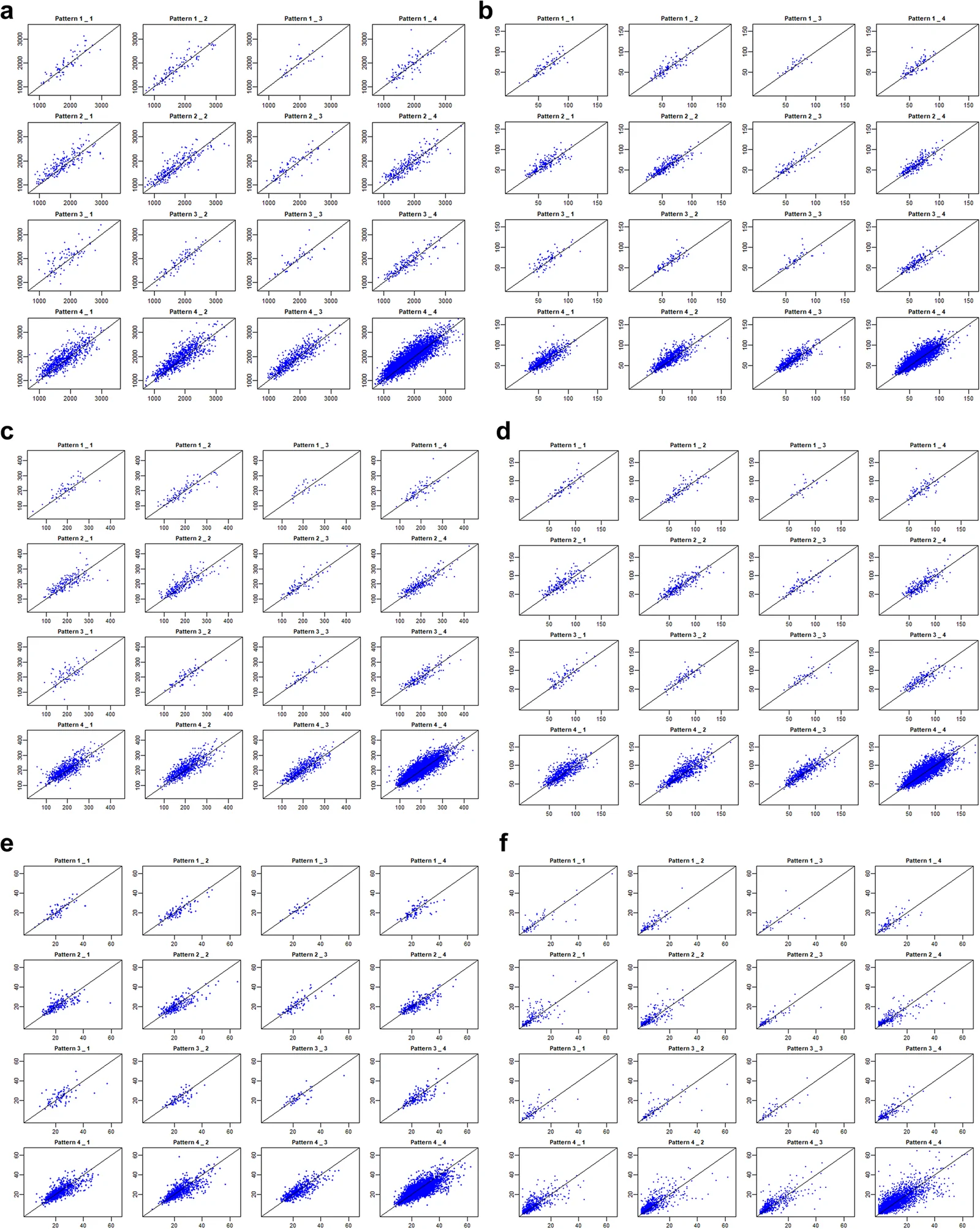
**a**. Scatterplots of Total energy intake estimates by available 24 h-FL pattern, re-examination (x-axis) vs. examination (y-axis). **b**. Scatterplots of Protein intake estimates by available 24h-FL pattern, re-examination (x-axis) vs. examination (y-axis). **c**. Scatterplots of Carbohydrate intake estimates by available 24 h-FL pattern, re-examination (x-axis) vs. examination (y-axis). **d**. Scatterplots of Fat intake estimates by available 24 h-FL pattern, re-examination (x-axis) vs. examination (y-axis). **e**. Scatterplots of Fiber intake estimates by available 24 h-FL pattern, re-examination (x-axis) vs. examination (y-axis). **f**. Scatterplots of Alcohol intake estimates by available 24 h-FL pattern, re-examination (x-axis) vs. examination (y-axis). Pattern A_B represents A 24h-FL available at baseline and B 24h-FL available at first re-examination

The scatterplots of total energy and macronutrient intakes at baseline and at first re-examination by patterns of available 24 h-FLs (Fig. 3a-f) show a very good agreement of estimates on this macronutrient level: for all patterns, observations are relatively narrow around the diagonal. The pattern names indicate how many 24 h-FLs were available for both time periods.

The scatterplots of usual intake estimates for the main food groups at baseline and first re-examination by pattern of available 24 h-FLs (Supplementary Fig. 1–14) showed this concordance to be less pronounced. As the aggregation level is lower, a greater variation in estimates between time periods was visible, especially for food groups that are consumed in smaller amounts, e.g., legumes (Supplementary Fig. 3), fish (Supplementary Fig. 8), or egg and egg products (Supplementary Fig. 9).

When limiting the sample to plausible reporters in the sensitivity analysis, we observed roughly the same mean estimate differences for the intake of total energy and energy-providing nutrients in both males and females (Table 4).

**Table 4:** Sensitivity analysis: median differences of re-examination -baseline estimates for plausible reporting status (0.8 ≤ Total energy intake/basal metabolic rate ≤ 1.6) and participants with full dietary recall available, e.g. four 24 h-FL in both examination waves

	Males	Females	Males	Females
	*N* = 3,806	*N* = 4,120	*N* = 2,552	*N* = 2,953
	**Plausible reporters**		**Availability of 24 h-FL: Pattern 4_4**	
Total energy intake [kcal/d]	-46.3	-39.4	-42.4	-31.7
Alcohol [g/d]	-0.3	-0.2	-0.1	-0.2
Fiber [g/d]	0.6	0.6	0.6	0.8
Protein [g/d]	-2.9	-1.7	-2.7	-1.6
Fat [g/d]	-1.4	-0.8	-1.1	-0.3
Carbohydrates [g/d]	-5.6	-6.0	-5.6	-5.5

## Discussion

In this study, dietary data from participants in two NAKO study centers (Berlin-South and Freiburg) that were collected during baseline and the first re-examination were analyzed to identify changes in dietary intakes between both time periods. Overall, good agreement of usual intake estimates at different levels and during both time periods was observed. The intake of total energy, macronutrients and main food groups differed only slightly, namely for meat and meat products and milk and dairy products. In addition, correlations between estimates during both time periods were high. These findings lead to the conclusion that no general change in diet has occurred in this group.

The data from this study were well-suited for our purpose. Most of the participants, namely over 60%, completed four 24 h-FLs in both examination waves. This information allowed the valid estimation of usual intake and thus also the assessment of changes in dietary behavior over time. The medians of the individual changes were around zero at the different levels of dietary information (macronutrients or food groups). This indicates that no substantial shift in a certain direction has occurred during that time span of nutritional assessment. Observed differences may as well reflect measurement error, which is a known problem in dietary assessment (see e.g. Gibson, Charrondiere [17]), although here, the same instruments are applied and therefore limiting this problem. The mean change in relation to the mean baseline estimate (percent change) is less than 5% for all macronutrients in males and females. Only for females did the mean alcoholic drinks intake decrease by 6%. Regarding food group intakes in males and females, the intake of meat and meat products decreased by 15% and 17%, respectively, although this is not reflected in the protein intake data. Less pronounced, also the intake of milk and dairy products decreased by 9% and 10% in males and females, respectively.

The stringent definition of non-consumers can contribute to the observed changes in meat and meat products intake, milk and dairy products intake and alcohol intake. Non-consumers were defined in the final step of the estimation process. Intake estimates were set to zero if all 24 h-FLs of a person did not indicate consumption of the specific food item and if, in addition, the FFQ indicated that a person never consumes that certain item. Evidently, those items indicated above are prone to radical changes, i.e., total avoidance, often propagated in social media or mainstream media or because of new-onset health problems. Although the median of the individual changes of intake was around zero, we acknowledge that some individuals have indeed adjusted their dietary intakes when looking at the tails of the distributions. Nonetheless, most participants did not substantially change their eating habits. A comparison of intake estimates derived for the full NAKO cohort showed that the identification of non-consumers as described above led to a lower total energy intake estimate; this was observed when comparing intake estimates from the full NAKO cohort with other studies, like the KORA FF4 study[5].

Due to the lack of dietary monitoring studies in Germany, no comparisons with data in the respective time frame can be made. The best studies for comparison are the KORA FF4, KORA Fit and the BVS 3 studies (18–20], as they cover a comparable calendar time. The KORA FF4 and KORA-Fit studies used a comparable methodology to estimate the usual dietary intake data. While no description of the change of intake between KORA FF4 and KORA Fit has been published, Rohm, Wawro [20] state that during the last 20 years, changes in food intake were observed (median processed meat intake reduction of 40%, decreased intake of dairy products, as well as increased vegetable and poultry intake). We expected to see less change in our data, as the time span is limited to about 5 years, which is a significantly shorter period; however, we can confirm a change in meat and dairy intake.

The comparison with available literature underlines the good level of agreement of usual intake data between both time periods in our study. Reproducibility over 4–5 years was also tested in other cohort studies, mainly showing moderate agreement in dietary intake variables (at the nutrient or food group level) [1, 2]. These studies often used FFQs. A study applying a comparable blended approach (as developed for the NAKO study) for which long-term reproducibility was tested could not be identified. On the other hand, Verbeke, Boedt [21] compared the results of a short web-based FFQ when repeated one week later (across several cohorts). They observed a Spearman correlation lower than in our study, where correlation coefficients (for macro-nutrients and total energy intake estimates) were even above 0.7. A recent review by Murai, Tajima [22] investigated the validity of web-based and conventional dietary assessment methods, such as dietary records or 24-h diet recalls. They reported acceptable correlations between estimates from different methods and supported a more widespread application of the web-based dietary assessment methods in the future.

A limitation of our study is that it was carried out in only two study centers. As the dietary assessment module was part of a very extensive examination program, the additional time for participants and study personnel to completely re-do the nutrition assessment at the first examination was only possible at the study centers in Freiburg and Berlin-South. Because the participating two study centers represent populations from the northern and the southern part of Germany, respectively, major dietary changes over time would likely be reflected within this combined sample. Additionally, limitations of the applied methodology should be mentioned. The FFQ and the 24 h-FLs provided a list of frequently consumed food items (plus the option “other”). Consequently, the questionnaires used did not cover ethnic foods or a broader range of vegetarian or vegan alternatives (for foods of animal origin). As amounts of consumption were not gathered by the questionnaires, it was necessary to use an external study population, namely the NVS II, to estimate consumption amounts. As there are no direct quantitative assessments of diet in the NAKO nor representative data on the current dietary habits of adults in Germany, the use of the NVS II data represents a shortcoming. Notably the 24 h-FL has not been formally validated against a gold standard. However, the consumption probability has greater impact on the variation in usual dietary intakes compared to the consumption amounts [23]. Furthermore, in our modelling, we did not account for temporal changes in adjustment variables, which would tend to underestimate dietary changes.

Remarkably, the inclusion of questionnaires from the first re-examination in estimating the usual intakes for the whole NAKO cohort made it possible to derive these intake estimates for 152,304 participants compared to 124,903 participants at the end of the baseline examination. In addition, the number of participants with four 24 h-FL filled out increased from roughly 22,500 to 71,500 participants, allowing more precise and valid modelling of the consumption probabilities [5]. Thus, the results presented here support the approach of pooling dietary assessment data collected during both examination waves to estimate usual intakes in the entire NAKO cohort. The absence of major changes in diet (with few exceptions) and the benefits of more valid probability modelling justify the approach to enhance data availability for NAKO participants with insufficient or missing dietary information at baseline.

## Conclusion

The results presented on the changes in usual dietary intake over 5 years within the NAKO cohort showed a high accordance at the food group and the macronutrient levels. Except for a decrease in the intake of meat and meat products and milk and dairy products, no major changes in dietary habits were observed. Ruling out substantial changes in dietary habits between these two time periods may therefore justify the approach of combining 24-FLs and an FFQ from both examination periods to derive usual intake estimates for a large number of participants in the full NAKO cohort in case of incomplete dietary data at baseline.

## Supplementary Information

Supplementary Material 1.

## Data Availability

The dataset supporting the conclusions of this article is not publicly available due to national data protection laws, since the informed consent given by NAKO study participants does not cover data posting in public databases. The dataset can be applied for via the German National Cohort (NAKO) transfer hub, https://transfer.nako.de, an electronic application portal. This data set was received under application No. NAKO-666.

## Abbreviations

NAKO: German National Cohort
NVS II: German National Nutrition Survey II
24 h-FL: 24 hour Food list
FFQ: Food frequency questionnaire
MSM: Multiple Source Method
SD: Standard deviation
ICC: Intraclass correlation coefficient
BMI: Body mass index
TEI: Total energy intake
BMR: Basal metabolic rate
