# Supplementary material for: Assessing the change in diet from baseline to first re-examination – results from the German National Cohort (NAKO)
Source: BMC Nutr. 2026 Aug 19;12:204. doi: 10.1186/s40795-026-01454-6 (PMC13633634; doi:10.1186/s40795-026-01454-6)
Supplement: Supplementary file 1 [file 40795_2026_1454_MOESM1_ESM.docx]

Supplementary Material

## 1.1 Supplementary Figures

**Supplementary Figure 1.** Scatterplots of Potatoes intake estimates by available 24h-FL pattern. Pattern A_B represents A 24h-FL available at baseline and B 24h-FL available at first re-examination.

**Supplementary Figure 2.** Scatterplots of Vegetables intake estimates by available 24h-FL pattern. Pattern A_B represents A 24h-FL available at baseline and B 24h-FL available at first re-examination.

**Supplementary Figure 3.** Scatterplots of Legumes intake estimates by available 24h-FL pattern. Pattern A_B represents A 24h-FL available at baseline and B 24h-FL available at first re-examination.

**Supplementary Figure 4.** Scatterplots of Fruits and Nuts intake estimates by available 24h-FL pattern. Pattern A_B represents A 24h-FL available at baseline and B 24h-FL available at first re-examination.

**Supplementary Figure 5.** Scatterplots of Milk and Diary products intake estimates by available 24h-FL pattern. Pattern A_B represents A 24h-FL available at baseline and B 24h-FL available at first re-examination.

**Supplementary Figure 6.** Scatterplots of Cereals intake estimates by available 24h-FL pattern. Pattern A_B represents A 24h-FL available at baseline and B 24h-FL available at first re-examination.

**Supplementary Figure 7.** Scatterplots of Meat and Meat products intake estimates by available 24h-FL pattern. Pattern A_B represents A 24h-FL available at baseline and B 24h-FL available at first re-examination.

**Supplementary Figure 8.** Scatterplots of Fish and Shellfish intake estimates by available 24h-FL pattern. Pattern A_B represents A 24h-FL available at baseline and B 24h-FL available at first re-examination.

**Supplementary Figure 9.** Scatterplots of Eggs and Egg products intake estimates by available 24h-FL pattern. Pattern A_B represents A 24h-FL available at baseline and B 24h-FL available at first re-examination.

**Supplementary Figure 10.** Scatterplots of Fats intake estimates by available 24h-FL pattern. Pattern A_B represents A 24h-FL available at baseline and B 24h-FL available at first re-examination.

**Supplementary Figure 11.** Scatterplots of Sweets and Sugar intake estimates by available 24h-FL pattern. Pattern A_B represents A 24h-FL available at baseline and B 24h-FL available at first re-examination.

**Supplementary Figure 12.** Scatterplots of Cake intake estimates by available 24h-FL pattern. Pattern A_B represents A 24h-FL available at baseline and B 24h-FL available at first re-examination.

**Supplementary Figure 13.** Scatterplots of Non-alcoholics drinks intake estimates by available 24h-FL pattern. Pattern A_B represents A 24h-FL available at baseline and B 24h-FL available at first re-examination.

**Supplementary Figure 14.** Scatterplots of Alcoholic-drinks intake estimates by available 24h-FL pattern. Pattern A_B represents A 24h-FL available at baseline and B 24h-FL available at first re-examination.
